# Supplementary material for: A sensor complements the steric gate when DNA polymerase ϵ discriminates ribonucleotides
Source: Nucleic Acids Res. 2023 Oct 11;51(20):11225–38. doi: 10.1093/nar/gkad817 (PMC10639073; doi:10.1093/nar/gkad817)
Supplement: gkad817_Supplemental_File [file gkad817_supplemental_file.pdf]

Supporting Information For:

A sensor complements the steric gate when DNA polymerase ε discriminates ribonucleotides

Vimal Parkash[1], Yashraj Kulkarni[2,3], Göran O. Bylund[1], Pia Osterman[1], Shina Caroline Lynn Kamerlin[2,4] and Erik Johansson[1,§]

[1] Department of Medical Biochemistry and Biophysics, Umeå University, Umeå, 90187, Sweden

[2] Department of Chemistry - BMC, Uppsala University, Box 576, Uppsala S-751 23, Sweden.

[3] Department of Drug Design and Pharmacology, University of Copenhagen, Universitetsparken 2, 2100 Copenhagen, Denmark

[4] School of Chemistry and Biochemistry, Georgia Institute of Technology, 901 Atlantic Drive NW, Atlanta, GA 30332-0400, USA.

[§] To whom correspondence should be addressed. Tel: +46 90 786 66 38; Email: erik.tm.johansson@umu.se

Table of Contents

Supplementary Figures 1-7……………..…………………………………………………………S3-S8

Supplementary Figure 1. Comparison of the polymerase active site in wild-type Pol2$_{CORE}$ and ^{M644G}Pol2$_{CORE}$ ternary complexes with a dNTP. S3

Supplementary Figure 2. Structural comparison of ^{M644G}Pol2$_{CORE}$-CTP and ^{M644G}Pol2$_{CORE}$-dCTP………………………………………………..……………………………………...S4

Supplementary Figure 3. A tetrad analysis shows that haploids with a *pol2-M644G,N828V* allele has a strong growth defect and that *rnh201Δ, pol2-N828V* strains are viable……………………………………S5

Supplementary Figure 4. Crystal structure of ^{N828V}Pol2$_{CORE}$ with an incoming dATP, superimposed on the structure of wild-type Pol2$_{CORE}$-dATP……………………………………......……………………S6

Supplementary Figure 5. N828 in Pol ε is a structurally conserved asparagine in B-family DNA polymerases.S7

Supplementary Figure 6. Box plots showing distribution of key distances and angles measured in MD simulations………………………………………………………………………………………S8

Supplementary Figure 7. Box plots showing distribution of metal-ligand distances (Å) during our simulations………………………………………………………………………………………S8

Supplementary Tables 1-9…………………………..……………………………………………S9-S13

Supplementary Table 1. Data collection and refinement statistics ………....………………………………..S9

Supplementary Table 2. Distance (Å) between C_α-atom of D877 and the OH group oxygen of Y645 during simulations of wild-type and M644G Pol ε in complex with dATP and ATP……………..……...……………..…S10

Supplementary Table 3. Distance (Å) between the 2'-C atom of the bound nucleotide (dATP/ATP) and the $C_{\delta1}$ atom of Y645 during simulations of wild-type and M644G Pol ε in complex with dATP and ATP……..…………………………………………………….....……………………………..S10

Supplementary Table 4. Angle (°) between the O2B atom of the dNTP and the C_α and C_γ atoms of the N828 side chain during simulations of wild-type and M644G Pol ε in complex with dATP and ATP…………….S10

Supplementary Table 5. Force field parameters for the tetrahedral Zn^{2+} dummy model used in this work ………....……………………………………………………………………………………S11

Supplementary Table 6. Metal-ligand distances (Å) between Zn metal center and its coordinating cysteine sidechain sulfur atoms calculated from out molecular dynamics simulations of wild-type and M644G Pol ε in complex with dATP and ATP…………………………………………………………………….S11

Supplementary Table 7. Spontaneous mutation rates ……………………....……………..…………………..…S12

Supplementary Table 8. Mutations found in *CAN1* gene of Can^{R} yeast strains……………………………...S12

Supplementary Table 9. Analysis of hydrogen bonds on which the "polar filter" model depends on………..S13

Supplementary References…………………………..………………………………………….S14

Supplementary Figures

Supplementary figure 1

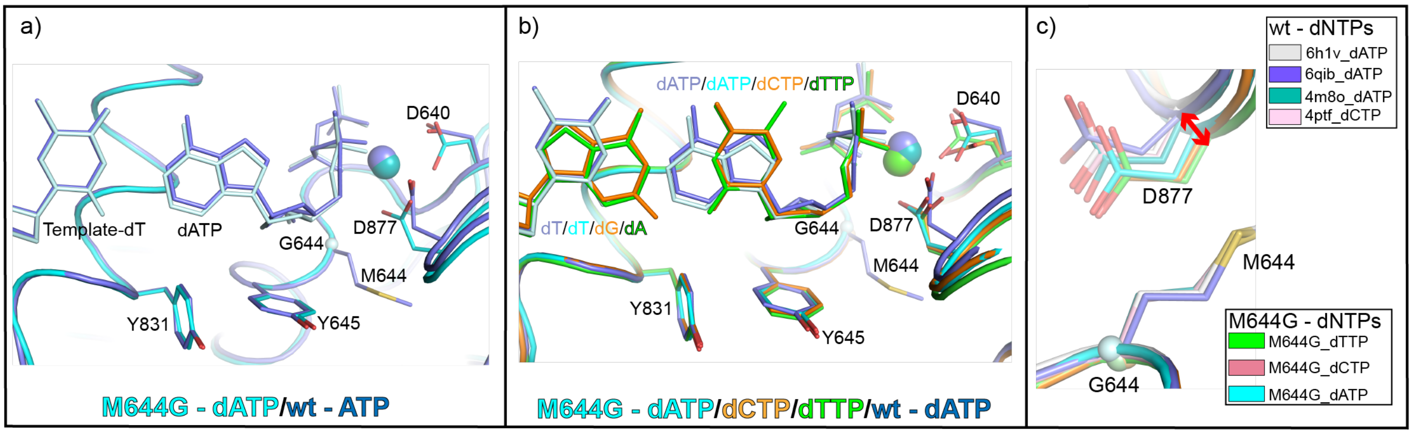

Supplementary Figure 1. Comparison of the polymerase active site in wild-type Pol2$_{CORE}$ and ^{M644G}Pol2$_{CORE}$ ternary complexes with a dNTP. a) The structures of ^{M644G}Pol2$_{CORE}$ (cyan) and wild-type Pol2$_{CORE}$ (blue) with dATP bound in the active site are superimposed. b) All ^{M644G}Pol2$_{CORE}$ structures containing dATP (cyan), dCTP (orange), or dTTP (green) are superimposed with the wild-type Pol2$_{CORE}$ (blue) structure. c) The structures of ^{M644G}Pol2$_{CORE}$ with different dNTPs (dATP, dCTP, or dTTP) are aligned to the available wild-type Pol2$_{CORE}$ structures (PDB IDs: 6h1v (1), 6qib (1), 4m8o (2), and 4ptf (3)). The loop containing D877 is slightly shifted (~ 1Å) in all ^{M644G}Pol2$_{CORE}$-dNTP structures when compared to the wild-type Pol2$_{CORE}$-dNTP structures.

Supplementary figure 2

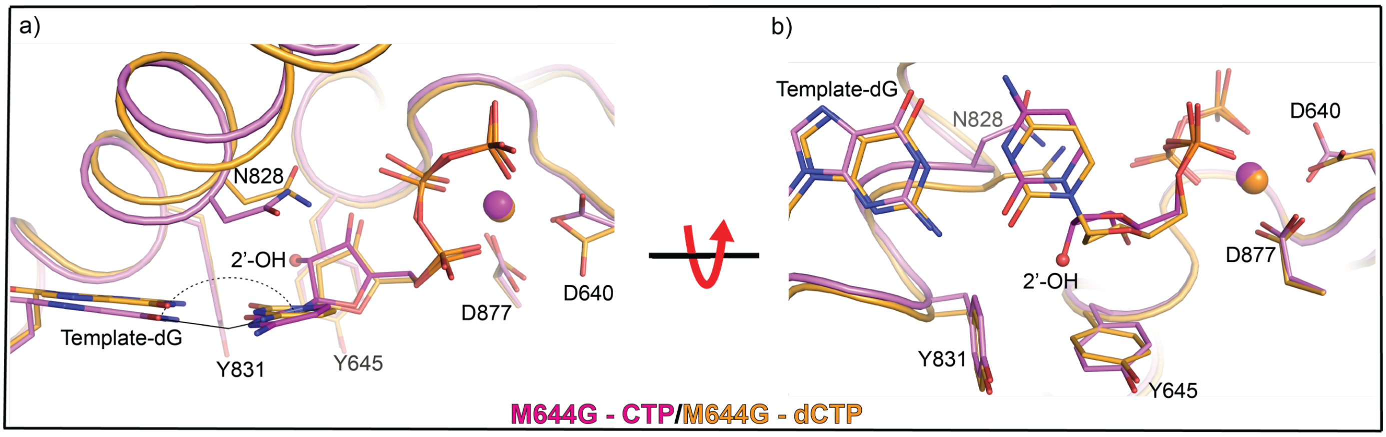

Supplementary Figure 2. Structural comparison of ^{M644G}Pol2$_{CORE}$-CTP and ^{M644G}Pol2$_{CORE}$-dCTP. a) Comparison of ^{M644G}Pol2$_{CORE}$-CTP (purple) and ^{M644G}Pol2$_{CORE}$-dCTP (orange) showing that the ribonucleotide takes a slightly different position compared to the deoxyribonucleotide. The nonplanar alignment with the templating base (dG) and CTP and the hydrogen bond between N828 and the beta-phosphate of dTTP are highlighted. b) The view is rotated by 90° along the horizontal axis to show the position of the 2'-OH group relative to the steric gate, Y645, and the shift of N828 in the finger domain.

Supplementary figure 3

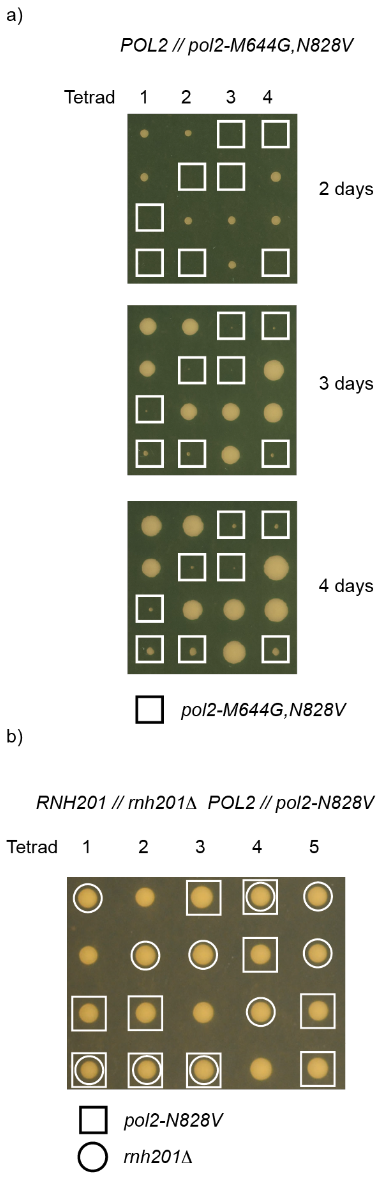

Supplementary Figure 3. Tetrad analysis showing that haploids with a *pol2-M644G,N828V* allele have a strong growth defect and that *rnh201Δ, pol2-N828V* strains are viable. a) *POL2/pol2-M644G,N828V* diploid yeast in the E134 strain background were sporulated followed by tetrad dissection. Colonies with cells carrying the *pol2-M644G,N828V* allele are in boxes. b) Diploid yeast, *RNH201/rnh201Δ, POL2/pol2-N828V* in the E134 strain background were sporulated followed by tetrad dissection.

Supplementary figure 4

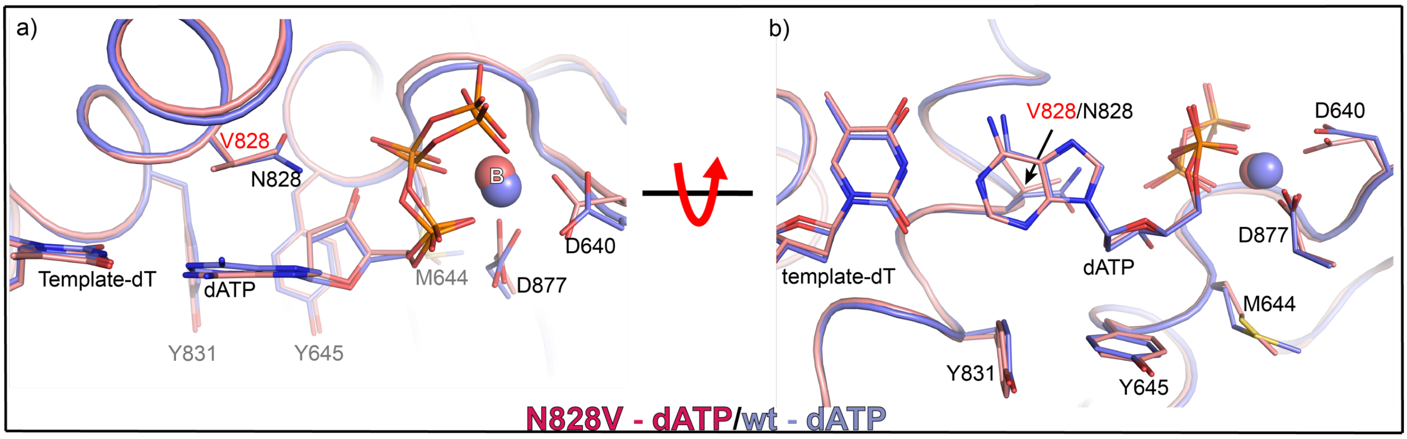

Supplementary Figure 4. Crystal structure of ^{N828V}Pol2$_{CORE}$ with an incoming dATP superimposed on the structure of wild-type Pol2$_{CORE}$-dATP. a) Comparison of ^{N828V}Pol2$_{CORE}$-dATP (in pink) and wild-type Pol2$_{CORE}$-dATP (in blue) showing that the deoxyribonucleotide had an identical position despite the N828V substitution in the finger domain. b) The view is rotated by 90° along the horizontal axis.

Supplementary figure 5

a)

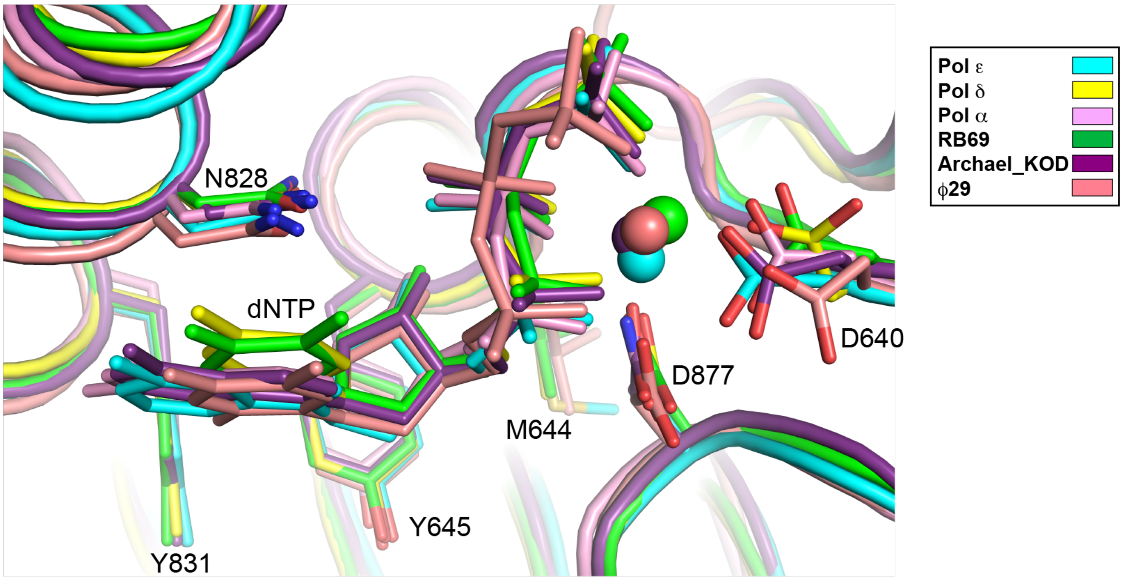

b)

Pol ε residue	640 / 644 645	828 / 831	877
Pol ε	Y H V D V A S M Y P N I M T	Q L A H K V I L N S F Y G Y	E L D T D G I W
Pol δ	A T L D F N S L Y P S I M M	Q L A L K I S A N S V Y G F	Y G D T D S V M
Pol α	L V M D F N S L Y P S I I Q	Q Q A L K L T A N S M Y G C	Y G D T D S V M
RB69	M S F D L T S L Y P S I I R	Q I N R K L L I N S L Y G A	Y G D T D S I Y
Archeal KOD	V Y L D F R S L Y P S I I I	Q R A I K I L A N S Y Y G Y	Y S D T D G F F
Pfu	V Y L D Y K S L Y P S I I I	Q K A I K L L A N S F Y G Y	Y I D T D G L Y
P. abyssi	V S L D F R S L Y P S I I I	Q R A I K I L A N S Y Y G Y	Y I D T D G L Y
SsPolB1	T V L D F A S L Y P S I I R	Q R A M K V F I N A T Y G V	Y G D T D S L F
ϕ29	M V F D V N S L Y P A Q M Y	Q L A - K L M L N S L Y G K	Y C D T D S I H

Supplementary Figure 5. N828 in Pol ε is a structurally conserved asparagine in B-family DNA polymerases. a) Superposition of structures from B-family DNA polymerases with a deoxyribonucleotide in the active site and a metal coordinated by catalytic aspartates in the B-site. b) Structure-based sequence alignment of B-family DNA polymerases highlighting the residues shown in the structural superposition (see panel a).

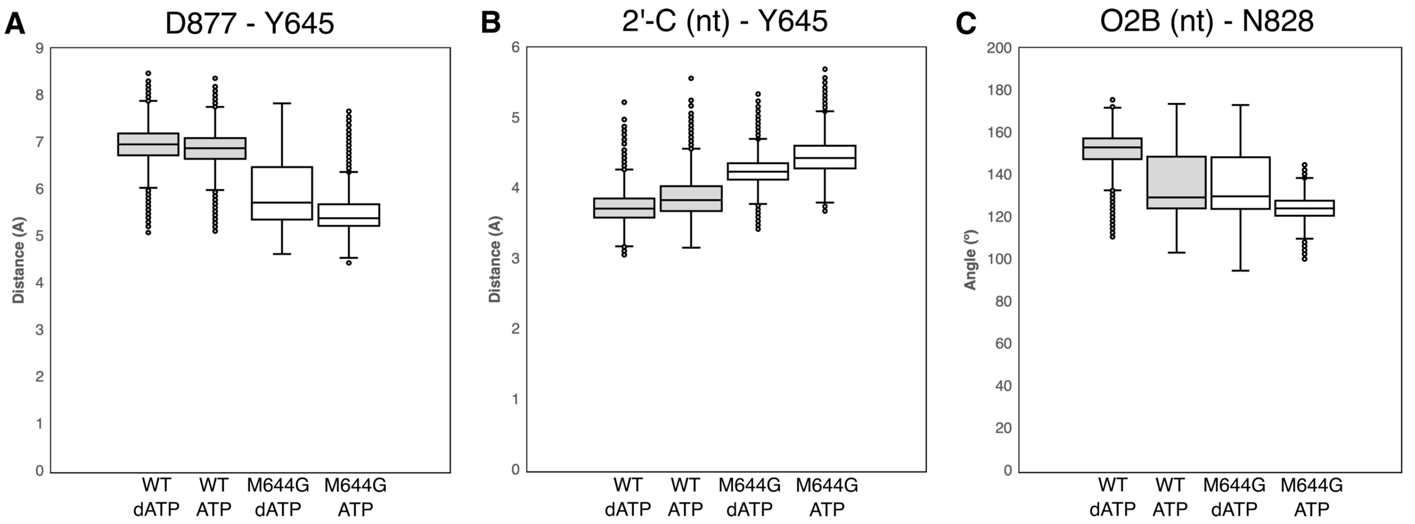

Supplementary Figure 6. **Box plots showing distribution of key distances and angles measured in MD simulations.** a) Distance between the C_{α}-atom of D877 and the OH-group oxygen of Y645. b) Distance between the 2’-C atom of the bound nucleotide (dATP/ATP) and the $C_{\delta 1}$ atom of Y645. c) Angle between the O2B atom of the nucleotide, and the C_{α} and C_{γ} atoms of the N828 side chain. All distances are in Å and angles in degrees (°). Shaded and white boxes correspond to wild-type Pol ε and M644G Pol ε respectively. Whiskers indicate standard deviations. Average and standard deviation values corresponding to panels a - c are provided in Supplementary Tables 2 through 4 respectively.

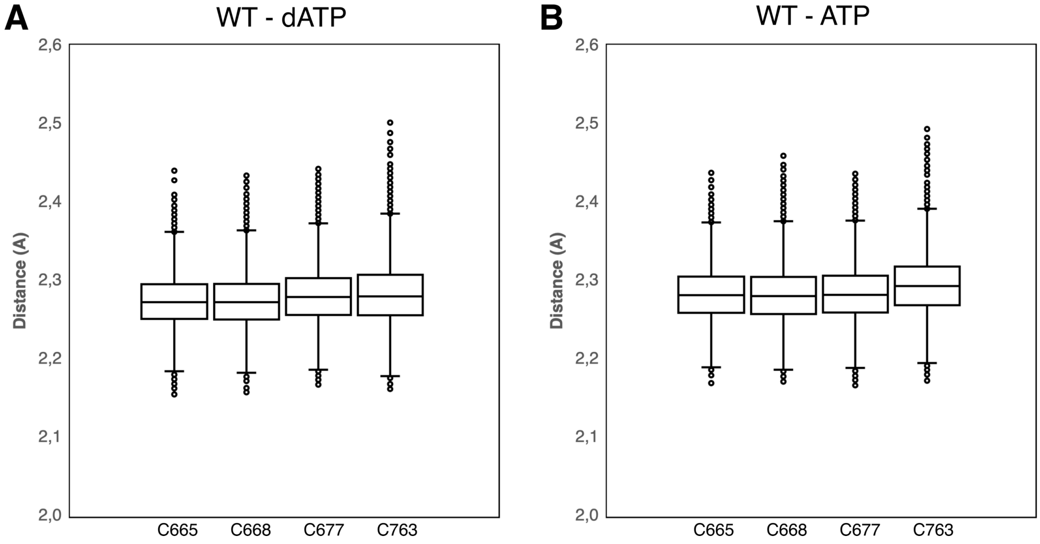

Supplementary Figure 7. **Box plots showing distribution of metal-ligand distances (Å) during our simulations.** Shown here are distances between the Zn metal center and its coordinating cysteine sidechain sulfur atoms, calculated from MD simulations of wild-type Pol ε in complex with a) dATP and b) ATP**.** Average and standard deviation values corresponding to panels a and b are provided in Supplementary Table 6.

Supplementary Tables

Supplementary Table 1. Data collection and refinement statistics

**PDB ID code**	8b76	8b6k	8b79	8b67	8b77	8b7e
Structure	M644G-dTTP	M644G-dCTP	M644G-UTP	M644G-CTP	N828V-dATP	N828V-UTP
**Data Collection**						
Spacegroup	P2	P2	P2	C2	C2	P2
Cell dimensions						
a(Å)	154.0	154.4	154.4	162.1	159.0	154.4
b(Å)	69.8	70.4	70.7	67.4	71.03	70.0
c(Å)	159.0	158.6	159.4	152.1	155.25	158.3
α, β, γ (°)	90.0, 112.6, 90.0	90.0, 112.7, 90.0	90.0, 112.8, 90.0	90.0, 111.5, 90.0	90.0, 113.0, 90.0	90.0, 112.6, 90.0
Resolution (Å)	2.60	2.50	2.65	2.60	2.70	2.60
R-merge (%)	9.2 (68.4)	20 (126)	12.7 (172.6)	14.9 (49)	12.3 (69.5)	16.6 (124.5)
I/sigmaI	7.4 (1.5)	5.4 (1.1)	9.9 (1.0)	5.6 (2.7)	4.5 (2.0)	6.8 (1.4)
Completeness	99.5 (99.8)	99.8 (99.1)	99.8 (99.9)	98.5 (99.8)	98.6 (99.7)	98.9 (99.9)
CC(1/2)	0.99 (0.42)	0.98 (0.08)	0.99 (0.46)	0.96 (0.40)	0.99 (0.42)	0.99 (0.32)
Redundancy	3.0 (3.1)	3.9 (3.6)	6.4 (6.6)	3.7 (3.9)	4.2 (4.4)	4.3 (4.4)
**Refinement**						
Resolution (Å)	2.6	2.5	2.65	2.6	2.7	2.6
Reflections	96114	109009	92507	46654	43315	95325
R_{work}/R_{free} (%)	22.0/24.6	22.9/26.0	21.7/24.8	23.9/27.1	23.9/27.2	20.0/23.0
TLS groups	-	7	-	11	6	19
Atoms						
Macromolecules	18761	18642	19007	9097	9293	18674
Ligands	72	58	72	36	31	68
H_2O	53	0	12	15	0	82
Average B-factors						
Macromolecules	64.4	59.9	76.6	56.3	83.3	58.2
Ligands	48.0	34.6	59.5	37.6	45.2	41.2
H_2O	47.1	-	65.6	36.7	-	43.6
RMSD						
Bond length (Å)	0.004	0.004	0.005	0.005	0.005	0.006
Bond angles (°)	0.69	0.66	1.003	0.88	0.86	0.88
Ramachandran						
Favoured (%)	96.2	96.1	96.0	96.7	94.7	96.3
Allowed (%)	3.7	3.9	4.0	3.3	5.3	3.6

Supplementary Table 2. Distance (Å) between the $C_α$-atom of D877 and the OH-group oxygen of Y645 during MD simulations of wild-type and M644G Pol ε in complex with dATP and ATP.[a]

**WT**		**M644G**	
**dATP**	**ATP**	**dATP**	**ATP**
7.0 ± 0.4	6.9 ± 0.4	5.9 ± 0.6	5.5 ± 0.4

[a] All distances are average values and standard deviations, averaged over 3 production replicas of 100 ns simulation time each, as described in the Materials and Methods, with snapshots taken every 12.5 ps for the analysis.

Supplementary Table 3. Distance (Å) between the 2'-C atom of the bound nucleotide (dATP/ATP) and the $C_{δ1}$ atom of Y645 during MD simulations of wild-type and M644G Pol ε in complex with dATP and ATP.[a]

**WT**		**M644G**	
**dATP**	**ATP**	**dATP**	**ATP**
3.7 ± 0.2	4.3 ± 0.2	3.9 ± 0.3	4.5 ± 0.2

[a] All distances are average values and standard deviations, averaged over 3 production replicas of 100 ns simulation time each, as described in the Materials and Methods, with snapshots taken every 12.5 ps for the analysis.

Supplementary Table 4. Angle (°) between the O2B atom of the dNTP and the $C_α$ and $C_γ$ atoms of the N828 side chain during simulations of wild-type and M644G Pol ε in complex with dATP and ATP.[a]

**WT**		**M644G**	
**dATP**	**ATP**	**dATP**	**ATP**
150.9 ± 10.5	135.0 ± 13.7	134.8 ± 13.8	124.5 ± 5.4

[a] All distances are average values and standard deviations, averaged over 3 production replicas of 100 ns simulation time each, as described in the Materials and Methods, with snapshots taken every 12.5 ps for the analysis.

Supplementary Table 5. Force field parameters for the tetrahedral Zn^{2+} dummy model used in this work.[a]

**Bonded Interactions**				
**Bond type**[a]		**K_b**		**r_0**
Zn-D		800.0		0.900
**Angle type**[b]		**K_θ**		**θ_0**
D-Zn-D		250.0		109.5
**Mass (*m*), Charge (*e*), and Non-bonded Interactions**[c]				
**Atom Type**	***m***	***e***	**A_i**	**B_i**
Zn	53.39	–1.00	68.00	38.00
D	3.00	0.75	0.05	0.00

[a] Zn represents the zinc metal center atom, while D represents the dummy atoms. [a] $U_b = K_b(b - b_0)^2$, where K_b is in kcal mol^{-1} Å^{-2} and r_0 is in Å. [b] $U_\theta = (1/2)K_\theta(\theta - \theta_0)^2$, where K_θ is in kcal mol^{-1} rad^{-2} and θ_0 is in degrees. [c] Lennard–Jones parameters are given in units of [kcal$^{1/2}$ mol$^{-1/2}$ Å^6] for A_i and [kcal$^{1/2}$ mol$^{-1/2}$ Å^3] for B_i.

Supplementary Table 6. Metal-ligand distances (Å) between the Zn metal center and its coordinating cysteine sidechain sulfur atoms calculated from our MD simulations of wild-type Pol ε in complex with dATP and ATP. Experimental distances for the corresponding atom pairs have been measured from the crystal structure coordinates of wild-type Pol ε (PDB: 4m8o).[a]

**Residue^{atom}**	**Crystal Structure**	**dATP**[a]	**ATP**[a]
$C665^{SG}$	2.3	2.3 ± 0.04	2.3 ± 0.04
$C668^{SG}$	2.3	2.3 ± 0.04	2.3 ± 0.04
$C677^{SG}$	2.2	2.3 ± 0.04	2.3 ± 0.04
$C763^{SG}$	2.4	2.3 ± 0.04	2.3 ± 0.04

[a] All distances are average values and standard deviations, averaged over 3 production replicas of 100 ns simulation time each, as described in the Materials and Methods, with snapshots taken every 12.5 ps for the analysis.

Supplementary Table 7. Spontaneous mutation rates.[a]

Genotype	CAN^r	
	**Mutation rate (x10^{-7})**	**Relative [a)]**
Wild-type	4.6 (4.0-5.9)	1
pol2-N828V	17.5 (14.4-20.1)	3.8
msh6Δ	16.6 (14.8- 25.5)	3.6
pol2-N828V msh6Δ	642 (553-916)	140

[a] Rates are relative to the wild-type strain. Measurements were performed as described in the Methods. Values in parentheses are 95% confidence intervals, calculated as described in ref. (4).

Supplementary Table 8. Mutations found in the *CAN1* gene of Can[R] yeast strains.

**Types of mutations**	**Number of mutations in the different strains**				**Rates (×10^{-8})**			
	**Wild type**	***Pol2-N828V***	***Msh6Δ***	***Pol2-N828V Msh6Δ***	**Wild type**	***Pol2-N828V***	***Msh6Δ***	***Pol2-N828V Msh6Δ***
Base substitutions:								
AT->TA	1[a]	74[b]	1	4	1	139	2	265
AT->CG	2	3	6	1	2	6	11	66
AT->GC	3	2	6	1	3	4	11	66
GC->CG	12	0	3	2	12	<1	5	132
GC->AT	7	5	52	55	7	9	92	3642
GC->TA	10	3	18	32	10	6	32	2119
–1 deletion	5	2	5	1	5	4	9	66
+1 insertion	2	2	1	1	2	4	2	66
Complex mutation	1				1	<1	<1	<1
Other	5	2	2	0	5	4	3	<1
Total	48	93	94	97				

[a] Data from Aksenova *et al*. (5). [b] 63 out of 74 substitutions were A->T, thus leading to an imbalance that could result from T-T mismatches during leading strand synthesis.

Supplementary Table 9. Exploring the existence of the possible hydrogen bonds on which the "polar filter" model depends (6). The analysis was performed using the H-bonds tool in ChimeraX(7).

Y-family Polymerase	PDB ID	Finger residue – O3' hydrogen bond[a]	Finger residue – beta phosphate hydrogen bond[a]
PolK (*H. sapiens*)	2oh2	Loose	None
Rev1 (*S. cerevisiae*)	2aq4	Loose	Strict
PolI (*H. sapiens*)	2dpj	None	Strict
Dpo4 (*S. solfataricus*)	4qw8	None	Strict
B-family polymerases			
Phi29 DNA Polymerase (phage)	2pyl	none (indirect via water molecule)	none (indirect via water molecule)
Pol δ (*S. cerevisiae*)	3iay	Loose	Loose
PolII (*E. coli*)	3k57	Loose	Loose
RB69 DNA polymerase (phage)	4fjl	Loose	Loose
Pol ε (*S. cerevisiae*)	4m8o	None	Loose
Pol α (*H. sapiens*)	4qcl	None	None

a) Here, we separated "strict" hydrogen bonds (fulfilling the precise geometric criteria for a hydrogen bond) from "loose" hydrogen bonds, identified with relaxed hydrogen-bond constraints (by 0.4 Ångströms and 20.0 degrees) indicating that tolerances have been applied to the precise geometric criteria. These standard criteria in ChimeraX came from a survey of high-resolution structures(8); empirically, tolerances of 0.4 Ångström and 20.0 degrees are considered to work well for most macromolecular structures. "None" indicates that ChimeraX could not find a hydrogen bond even with the relaxed hydrogen-bond restraints.

Supplementary References

1. Ter Beek, J., Parkash, V., Bylund, G.O., Osterman, P., Sauer-Eriksson, A.E. and Johansson, E. (2019) Structural evidence for an essential Fe-S cluster in the catalytic core domain of DNA polymerase. *Nucleic Acids Res*, **47**, 5712-5722.
2. Hogg, M., Osterman, P., Bylund, G.O., Ganai, R.A., Lundstrom, E.B., Sauer-Eriksson, A.E. and Johansson, E. (2014) Structural basis for processive DNA synthesis by yeast DNA polymerase ε. *Nat Struct Mol Biol*, **21**, 49-55.
3. Jain, R., Rajashankar, K.R., Buku, A., Johnson, R.E., Prakash, L., Prakash, S. and Aggarwal, A.K. (2014) Crystal structure of yeast DNA polymerase epsilon catalytic domain. *PLoS One*, **9**, e94835.
4. Shcherbakova, P.V. and Kunkel, T.A. (1999) Mutator phenotypes conferred by MLH1 overexpression and by heterozygosity for mlh1 mutations. *Mol Cell Biol*, **19**, 3177-3183.
5. Aksenova, A., Volkov, K., Maceluch, J., Pursell, Z.F., Rogozin, I.B., Kunkel, T.A., Pavlov, Y.I. and Johansson, E. (2010) Mismatch repair-independent increase in spontaneous mutagenesis in yeast lacking non-essential subunits of DNA polymerase epsilon. *PLoS genetics*, **6**, e1001209.
6. Johnson, M.K., Kottur, J. and Nair, D.T. (2019) A polar filter in DNA polymerases prevents ribonucleotide incorporation. *Nucleic Acids Res*, **47**, 10693-10705.
7. Pettersen, E.F., Goddard, T.D., Huang, C.C., Meng, E.C., Couch, G.S., Croll, T.I., Morris, J.H. and Ferrin, T.E. (2021) UCSF ChimeraX: Structure visualization for researchers, educators, and developers. *Protein Sci*, **30**, 70-82.
8. Mills, J.E. and Dean, P.M. (1996) Three-dimensional hydrogen-bond geometry and probability information from a crystal survey. *J Comput Aided Mol Des*, **10**, 607-622.
